# Teaching Multilingual Students During the COVID-19 Pandemic in Austria: Teachers’ Perceptions of Barriers to Distance Learning

**DOI:** 10.3389/fpsyg.2022.805530

**Published:** 2022-03-10

**Authors:** Marie Gitschthaler, Elizabeth J. Erling, Katrin Stefan, Susanne Schwab

**Affiliations:** ^1^University College of Teacher Education of Christian Churches Vienna/Krems, Vienna, Austria; ^2^Institute of Multilingualism, Karlsruhe University of Education, Karlsruhe, Germany; ^3^Department for English and American Studies, University of Vienna, Vienna, Austria; ^4^Centre for Teacher Education, University of Vienna, Vienna, Austria; ^5^Optentia Research Focus Area, North-West University, Vanderbijlpark, South Africa

**Keywords:** COVID-19 pandemic, multilingual students, barriers to distance learning, language support, teachers’ perceptions, qualitative study

## Abstract

Providing high-quality education for students with emergent proficiency in the language of instruction (referred to here as multilingual students) presents a challenge to inclusion for educational systems the world over. In Austria, a new German language support model was implemented in the school year 2018/19 which provides language support in separate classrooms up to 20 h a week. Since its implementation, the model has been strongly criticized for excluding multilingual students from the mainstream classroom, which is argued to reinforce the educational disadvantages that they face. The study presented here provides unprecedented qualitative insight into how schooling for students within the so-called German language support classes (GLSC) was organized during the COVID-19 pandemic. It builds on results of a previous large-scale quantitative study (*n* = 3,400 teachers), which was conducted during the first lockdown (spring 2020) and indicated a high risk of exclusion for marginalized students, especially for multilingual students in GLSC. To gain deeper insights into the situation of these students during school closures, 37 teachers who work in these classes at both primary and lower-secondary schools in Vienna were interviewed, of which 18 interviews were considered for analysis. The interviews focus on the situation during the first and second school closures in the city of Vienna. A thematic analysis of the interview data reveals teachers’ perceptions of aspects which harmed or promoted inclusion for students in GLSC during these periods of school closure. Teachers’ perceptions of the most harming factors for students included strong language barriers between teachers and students, restricted access to technical equipment and supportive learning spaces, and low parental engagement. A development that promoted inclusion of these students was the option to allow them to come to school during the second school closure. Since existing studies on the schooling of students during school closures have hardly addressed the situation of students in GLSC, this study contributes to closing this research gap.

## Introduction

According to the Eurydice Report “Integrating Students from Migrant Backgrounds into Schools in Europe” (European Commission et al., 2019), the majority of European countries consider the provision of high-quality language and learning support for students with immigrant background as the greatest challenge. Because of major international voluntary and forced immigration movements, many education systems were already struggling to meet the goals of inclusion set in the Sustainable Development Goals (United Nations, 2015). Providing high-quality language support is a fundamental aspect of including students with an immigrant background in education and ensuring equal access to learning in the European Union (European Commission et al., 2019). Different pedagogical approaches for supporting students to learn the language of instruction have been developed, not all of which necessarily follow evidence-based good practice in language education (see Erling et al., 2022). An example of this is the new model of German language support classes (GLSC) developed in Austria, which was implemented in the school year 2018/19. This model, implemented without piloting nationwide, provides language support in separate classrooms for students with beginning or emergent competences in the language of instruction for up to 20 h of the 26–34-h school week. In the public discourse, these students are often referred to as having “a non-German colloquial language” – a term that suggests that they cannot speak German. However, many of these children have acquired German skills in the course of their socialization in informal contexts and in formal pre-school education, and/or use German, among other languages in their everyday lives. We therefore refer to this population as “multilingual students,” in recognition of the valuable resource of their language skills. Drawing on Collier and Thomas (2017), we also use the term “students with beginning or emerging skills in the language of instruction” or simply refer to multilingual students who attend GLSC.

When schools closed around the world in March 2020 due to the COVID-19 crisis, numerous studies (e.g., Kim and Asbury, 2020; Letzel et al., 2020; Schleicher, 2020; Helm et al., 2021) appeared in a relatively short period of time that examined the impact of distance learning on students, teachers, parents and other education stakeholders. The meta-study by Helm et al. (2021), who reviewed 97 studies on the impacts of school closures provide insight into the situation in the German-speaking world. The study confirmed that the situation of home-based learning had exacerbated existing educational inequalities and that the voices of the ‘hard to reach’ were often not included in research (Helm et al., 2021).

To gain more insights into home-based learning and development of students facing various types of educational disadvantage, including students in GLSC or with special education needs, Schwab and team conducted a large-scale quantitative study with teachers in Austria. Findings indicated a high risk of exclusion for students already at risk of marginalization, including those in GLSC (Schwab and Lindner, 2020; Kast et al., 2021). Moreover, many teachers in GLSC or special classes reported that it was nearly impossible to stay in contact with their students during that time (see Kast et al., 2021).

Equipped with the quantitative evidence collected in the first stage of the pandemic, a follow-up, mixed-method study (“Inclusive Home Learning in German language support classes”) was conducted to provide deeper insights into how schooling for students within GLSC was organized during the first and second school closures in Vienna, Austria. For the qualitative part of the study reported on here, 37 teachers who work in these classes at both primary and lower-secondary schools in Vienna were interviewed following the principles of problem-centered interviews (Witzel and Reiter, 2012). Thematic analysis focused on factors that were perceived as being harmful to inclusion as well as those offering potential to promote inclusion. Findings indicated that the teachers perceived the language barriers between them and their students as the strongest barriers in facilitating distance learning for multilingual students. Further, they referred to the students’ restricted access to technical equipment and supportive learning spaces at home, as well as the low engagement of their parents. The change in regulations for the second school closure, namely to allow students from GLSC and students with special educational needs to come to school, was perceived as an important factor in ensuring their access to education.

The motivation to conduct this study is based on two main rationales: Firstly, the authors have been researching GLSC since their introduction and have already conducted a large-scale study (Gitschthaler et al., 2021; Resch et al., forthcoming), the results of which indicate that the separate schooling of multilingual students has negative effects on their academic, social and emotional development. Therefore, it was a particular concern to explore how these children fared during the school closures. Secondly, existing studies on the schooling of students during COVID-19 have only minimally focused on students who are still learning the language of education. This article provides unprecedented insights into how GLSC might harm or promote inclusion, how this potential was affected by the COVID-19 pandemic, and how these insights might shape the development of GLSC going forward.

### German Language Support Classes in Austria

As in most European Union Member States, students in Austria with no, beginning or emergent skills in the language of instruction receive support in language support classes (cf. European Commission et al., 2019). These students should learn the national language of instruction as quickly as possible in order to participate more successfully in mainstream education (BMBWF, 2019). After many years of integrative, partly integrative or partly segregative language learning, the German language support program was reformed in the school year 2018/2019. Following this, a new, un-tested model of segregative language education that goes against “good practice” guidance (cf. European Commission et al., 2019) was rolled out across Austria. Policy stipulated that students with no or beginning German language skills were to attend separate classes for either 15 h per week (of the ca. 21-h school week) in primary school or 20 h in secondary school, which is a significant period of the ca. 35-h school week. Students with emergent German language skills attend the mainstream classroom most of the time, but get pulled out for 6 h per week to receive additional German language support. The decision of whether students have to attend GLSC is based on the results of a standardized assessment called the “MIKA-D,” which was designed to measure young people’s competences in German.

The recommended scenario is followed for one school year but can be extended to 2 years depending on the student’s results. After the end of each semester, students are given the same standardized test, with the results determining further transitions. While students following scenario 2 who improve their scores transfer into mainstream classes, students following scenario 3 either transfer into pull-out courses or mainstream classes, depending on their results. If students’ results do not improve, they remain in the specific support model for at least another semester^1^. However, if they do not transition to mainstream classes after two semesters, students have to repeat the grade.

Proponents of GLSC argue that segregated courses give students more time and space to learn the language of instruction outside the regular classroom. Teachers in GLSC can better adopt the lessons according to the learners’ needs and refer to their previous knowledge and skills (Jeuk, 2015). Segregated courses are deemed particularly necessary at the secondary level, where the accelerated learning of the language of instruction is especially necessary, so that students can keep up with the increasing complexity of content (Koehler, 2017).

Despite these arguments in favor of segregated language classes, their introduction has been widely criticized by applied linguists and educational researchers in Austria, who argue that this model goes against evidence-based notions of language education as well as the promotion of inclusion and emotional wellbeing (e.g., Müller and Schweiger, 2018; Döll, 2019). Critics of GLSC point to the large number of studies that have shown that the best language support models provide integrated, sustained, language support, ideally from the pre-school to the upper-secondary level (e.g., Collier and Thomas, 2017; Crul, 2017; Erling et al., 2022). Being pulled out of mainstream courses often has adverse effects on students’ progress in other academic subjects (Jeuk, 2015; Bunar, 2017; Herzog-Punzenberger et al., 2017). Moreover, in segregated language courses, learners are largely denied contact with peers who are more proficient in the language of instruction. Further concerns have been expressed regarding the social integration of these students, who often get stigmatized as being “remedial” (Ovando et al., 2005, p. 73; Karakayali et al., 2016).

While a formal evaluation of the current GLSC has yet to be undertaken, statistics about students’ transition to the mainstream classroom suggests that they are ineffective. One year after introducing GLSC (at the end of the 2018/2019 school year), only around 32% of the students made the transition to mainstream classes, while about half (48%) were transferred to pull-out courses (Scenario 2). Around 16% continued in the GLSC (Scenario 3) and 4% dropped out of the school system all together (Statistik Austria, 2019). Further evidence of the failure of GLSC to provide inclusive language education can be gleaned from a large-scale, quantitative study (including open-ended questions) that explored teachers’ perceptions (*n* = 1,267) of the positive and negative effects of GLSC during its first year of implementation (Gitschthaler et al., 2021; Resch et al., forthcoming). The main finding of the quantitative part of the study is that a segregative language support model, like the GLSC, was rated as rather positive or positive for the academic, social and emotional development of multilingual children by about 36% of the participating teachers. In contrast, an inclusive model was rated as rather positive or positive by 55.5% of teachers, respectively (Gitschthaler et al., 2021). Within the open-ended questions, 714 teachers reported negative effects of GLSC on an organizational, didactic and social dimension: While 50% of these teachers (*n* = 357) reported that GLSC further enhance segregation and exclusion of multilingual students, more than one fifth stated that the classes were under-resourced and that class sizes were too large (often having between 14 and 27 students). Hence, teachers reported being overworked and overwhelmingly unable to handle the heterogeneity of groups regarding diversity of language backgrounds, German competence and literacy levels (Resch et al., forthcoming).

Given that inclusion of multilingual students and their language learning were already perceived as being severely challenged by the model of GLSC in Austria, there were valid reasons for concern when teachers and students in these courses had to adapt to home-based schooling during the COVID-19 pandemic.

### Schooling During the COVID-19 Pandemic in Austria

In response to the COVID-19 pandemic, schools in Austria were closed several times, with various levels of openness depending on the severity of the pandemic, the level of schooling and the availability of testing and vaccinations. During all closures, schools remained open for children of key workers, and increasingly also as the pandemic endured, for vulnerable children. The first and most severe school closure lasted from March until May 2020, with the majority of students staying home during this time and only returning to school in shifts in mid of May 2020 until the end of the school year in July 2020 (BMBWF, 2020a). The second school closure lasted from November until December 2020, with students returning in shifts for a short period before the Christmas holidays. The third school closure was essentially a continuation of the Christmas holidays in January 2021 (BMBWF, 2021a). From February until May 2021, there was a fourth stage in which students from elementary and special schools went back to school. In secondary schools, classes were divided in half and students returned in shifts (BMBWF, 2021b). Because of evidence pointing to particularly harmful risks of school closures for students ‘at risk’ (e.g., those in GLSC and/or with specific educational needs), the regulations for school closures from November 2020 were changed and they were encouraged to return to school (BMBWF, 2020b).

From the first school closure in March 2020 onwards, teaching mostly moved to home-based, digital settings. A number of researchers rapidly responded to school closures and designed studies investigating the educational experiences of students, teachers and other education stakeholders. In the German speaking world, the School Barometer monitored the school situation by collecting the perspectives of various actors (i.e., parents, students, school staff, school leadership, school authority, and school support system) and contributing a data-informed discussion about various aspects of teaching and learning (e.g., Huber and Helm, 2020; Helm et al., 2021). Furthermore, Helm et al. (2021) conducted a meta-study of 97 studies conducted in Austria, Germany and Switzerland during the COVID-19 pandemic. Overall, the study confirmed that the situation of home-based learning had exacerbated existing educational inequalities. However, it also pointed to disruptions from the ‘normal’ schooling routine that may have inadvertently had a positive impact on students.

With regard to the provision of online education, the findings indicated that the majority of students did not face limiting issues with regard to access to ICT and the vast majority of teachers were found to be delivering content. Both analog and digital media were used, the most common approach being using the textbooks (Albaner et al., 2021). In some schools, especially primary schools, students received weekly learning plans and materials that could be picked up at school (cf. Helm et al., 2021). Learning materials were also provided via digital tools like e-mail or learning platforms. In many contexts, online teaching was provided using video conferencing systems like Teams or Zoom.

Since home-based learning had to be implemented very quickly (e.g., Bozkurt et al., 2020; Reimers et al., 2020), it initially caused uncertainty and a lack of understanding among many teachers who received information only at the last minute through public news channels (Schwab and Lindner, 2020). As a result, teachers were required to have a high degree of flexibility, adaptability and technical knowledge (König et al., 2020; Tengler et al., 2020). Unsurprisingly, teachers in Austria felt (rather) heavily stressed during this period (Schwab and Lindner, 2020). Despite this, they were found to have coped well with the transition to home-based learning and were confident in their abilities to teach their subjects adequately (Schober and Holzer, 2020). However, findings of several studies found that home-based learning only worked well for students who did not face serious obstacles and that for a minority of students (which makes up a substantial number in real terms), education was extremely limited during the home-based learning period.

In this context, the large-scale study “INCLusive home LEArning” (INCLEA) on the consequences of home schooling during the first lockdown provides important insights in the situation of socially disadvantaged students. More than 3,400 teachers participated in the online survey and were asked for their perceptions of the effect of the situation on their students and their learning. Findings confirmed fears and indicated a high risk of exclusion for students already at risk of marginalization, including those in GLSC (see Schwab and Lindner, 2020; Kast et al., 2021). Evidence from other studies underpin these results. Steiner et al. (2021) found that teachers were not at all able to reach 36% of socially disadvantaged students. This may be due to the often-reported lack of adequate endowments with technical equipment and digital skills. Holtgrewe et al. (2020) showed that 13% of students have difficulties using a computer, 20% have difficulties with online meeting tools, and 37% reported difficulties using learning platforms. Access to ICT was also a problem for some students from low SES backgrounds: 3% were found not to possess a computer; 6% not to have a quiet learning environment at home; 8% to have limited access to a computer; 1% not to have an internet connection; 5% only use their smartphone for home-based learning; and 25% share their computer with others (Holtgrewe et al., 2020).

Further risks arise from non-individualized ‘learning packages,’ which pose great difficulties for students from socially disadvantaged families in particular. Schwab et al. (2020) found that over 31% of teachers reported not individualizing ‘learning packages’ based on their students’ knowledge. While teachers may have suspected that a large number of students did not receive the necessary support to complete assignments at home [an estimated 40% in Steiner et al. (2021)], a large number of them reported not being able to compensate for this in their teaching approaches.

Taken together, these studies conducted all allude to factors which may have contributed to the limited participation of some students, including restricted access to and competence using technology; a lack of pedagogical concept in the materials delivered, particularly with regard to students from low SES or/and multilingual backgrounds; and limited emotional and pedagogical support available at home. Thus, it seemed very likely that the particular situation of enforced home-based learning would exacerbate the risks to inclusion for students in GLSC – who were already deemed as being poorly served by this model before the pandemic.

Apart from the “Inclusive Home Learning” study (see Schwab and Lindner, 2020; Kast et al., 2021), to the best of our knowledge, no extensive studies have investigated the specific situation of multilingual students in GLSC during school closures. Furthermore, most studies have applied a quantitative approach, not giving enough space to the voices of participants. This qualitative study thus contributes to closing these research gaps and offers insights into the learning situation of these students from the perspective of teachers who teach in GLSC.

### Empirical Study

Equipped with the quantitative evidence collected in the first stage of the pandemic, the study reported on here sought to provide deeper insights into the experiences of multilingual students in GLSC during school closures. Based on the findings from “Inclusive Home Learning,” a spin-off, mixed-method study (“Inclusive Home Learning in German language support classes”) was conceived in order to more thoroughly investigate the situation of students in GLSC. The quantitative part of the study comprises a sample of 2,651 teachers working in regular classes and in GLSC at primary, middle and special schools from all over Austria. The items of the questionnaire referred to teachers’ perceptions regarding students’ burden, the provision of weekly individual coaching for students, the students’ opportunities to work with digital devices; further items referred to the teachers’ perception of students working actively at home and whether distance learning increases educational disadvantages. The most important findings that shaped the set-up of the qualitative aspect of the study were that almost 40% of GLSC teachers perceived their students to be less actively working on their school tasks. In contrast, this was reported by only about 9% of teachers in regular classes. With regard to digital devices, 32% of GLSC teachers perceived that their students had no opportunity at all to use a computer or a tablet for schoolwork. Again, this value was much lower for teachers in regular classes (4.6%) (Lindner et al., forthcoming). The results indicated great difficulties in implementing distance learning for students in GLSC. By giving voice to GLSC teachers, we aimed at getting deeper insights into how schooling for students within GLSC was organized during school closures: 37 GLSC teachers at both primary and lower-secondary schools in Vienna were interviewed and 18 interviews were included for analysis. The research questions driving the qualitative part of the study were:

• How did teachers provide learning opportunities for their students in GLSC during the home-based learning periods?
• What did GLSC teachers perceive as the greatest barriers to reaching their learners during this time?
• What, if any, strategies did they develop for promoting inclusion despite the significant obstacles they faced?

In exploring these questions, this article contributes unprecedented insight into how GLSC might harm or promote inclusion, and how this potential was affected by the COVID-19 pandemic.

## Materials and Methods

Semi-structured interviews were conducted with teachers in GLSC following the principles of the problem-centered interview method (Witzel, 2000; Witzel and Reiter, 2012). For this study, this meant that the researchers were orientated toward a socially relevant problem (i.e., the situation of GLSC students during home-based learning) about which they had prior knowledge. In this case, the researchers’ prior knowledge was obtained through the quantitative research they were involved (see Schwab and Lindner, 2020; Kast et al., 2021), and the wealth of publications about schooling during the pandemic mentioned above. While this study sought to provide insight into students’ experiences, teachers were interviewed instead, as the challenges that students faced with regard to language, access to technology, etc., made it difficult for them to take part in online research. The study sought insights into teachers subjective perspectives of how they and their students experienced home-based learning, and these teachers were considered to be “experts of their orientations and actions” (Witzel, 2000).

Results from the quantitative part of this study (“Inclusive Home Learning in German language support classes”) provided a “heuristic framework” for the development of the interview schedule. The questions were formulated in general terms to avoid determining teachers’ answers. Throughout the interviews, participants were given sufficient space to express their experiences and subjective perspectives (Witzel and Reiter, 2012, p. 4). As a narrative-generating introductory question, the interviewees were asked to think back to March 2020 - the beginning of home-based learning - and how they remember this time as a teacher of GLSC. The teachers’ descriptions structured the further thematic course of the interview and provided the interviewers with opportunities to address the following additional topics: Organizing students’ learning; students’ barriers to learning; biggest challenges in daily work; differences between the first and second school closure; effects of home-based learning on language acquisition and conditions for successful language acquisition in distance learning.

### Data Collection

The data collection for this study was organized by the third author with the support of five students (see section “Acknowledgments”). The interviews took place in December 2020 (i.e., the period of the second school closure in Austria) in Vienna. The decision to focus on Vienna was based on official statistics that show that the proportion of students who have German as an additional language in primary schools is considerably higher in Vienna (58.5%) compared to the other federal states of Austria (average: 23.4%) (Statistik Austria, 2021a).

Following a purposeful sampling strategy (Patton, 2015), 37 participants were selected, based on the main criterion that they worked in GLSC during both school closures at primary schools and middle schools in Vienna. This ensured that they had specific knowledge based on their role as the teachers of these classes and therefore could be considered as information-rich cases. Contact was established through direct contact with schools by email and telephone, but also through social media platforms.

All interviews were conducted in German using video conferencing software, e.g., Zoom or MS-Teams. The interviews lasted between 45 and 149 min (*M* = 67.3, *SD* = 18.6) and were transcribed in full length by following the transcription conventions of Kuckartz (2018).

### Sample

While interviews were conducted with 37 teachers, in the process of analysis, the researchers soon discovered that not all teachers interviewed worked in GLSC during both school closures. This was problematic since differences between the first and second lockdown were of key interest for this study. Therefore, these interviews were not further considered in the analyses. Further, the experiences of teachers in middle schools were mostly not comparable to those in primary schools due to differences in students’ age as well as language and digital skills. To allow comparability of the data, interviews with middle school teachers were also excluded from the analysis. Consequently, 18 interviews with primary school teachers who worked in a GLSC during both lockdowns remained in the data set (see Table 1).

**TABLE 1:** Sociodemographic characteristics of the 18 participants.

ID	f/m	Age	First language(s) spoken	Years of teaching experience	Years of teaching in GLSC	Training in teaching German as L2	Number of grade levels in GLSC	Number of students in GLSC
T_1	f	26	German	4	3	No	4	19
T_2	f	32	German	5	3	Yes	4	17
T_3	f	23	German	2	1	No	1	10
T_4	f	62	Hungarian	40	1	No	5	14
T_5	f	34	German	5	2	No	3	9
T_6	f	27	German	1	0,5	Yes	2	10
T_7	f	55	German	25	3	Yes	1	8
T_8	f	44	German	20	3	Yes	4	13
T_9	f	52	German	10	3	Yes	3	14
T_10	f	26	German	2	2	Yes	3	12
T_11	f	40	German	14	3	No	3	15
T_12	f	41	German	19	3	Yes	4	14
T_13	f	45	German	8	2	Yes	4	11
T_14	f	27	German	2	1	No	3	10
T_15	f	26	Turkish	5	3	No	1	20
T_16	f	43	German	4	3	Yes	4	8
T_17	f	59	German and English	20	3	Yes	2	12
T_18	f	25	German	1	1	No	1	18

All of the teachers were female, which is not surprising given that ca 92% of all primary teachers are female (Statistik Austria, 2021b). The mean age of the participants was 38, and the range was 23–62 years of age. The mean years of teaching experience was 10, with the range being 1–40 years. As GLSC were recently introduced, teachers’ years of teaching in them varies from 1 to 3 years.

Only 3 of the 18 teachers indicated that they have another first language than German or were raised bilingually. About half of the teachers had some kind of education in Teaching German as Second or Foreign language. Since such programs have only recently been introduced, teachers show a range of teacher education opportunities, which do not always follow a standardized curriculum and are, therefore, not equally valued and recognized.

The grade levels ranged from grade 1 to grade 4 of primary school, which means that the students were between 6 and 10 years old. The classes are thus characterized by a high degree of heterogeneity in terms of students’ knowledge and language abilities. The number of students in each teacher’s class varied between 8 and 20.

### Ethical Considerations

Permission to contact schools and conduct interviews with teachers was obtained from the Board of Education for Vienna. Once contact to teachers was established with the support of schools’ head teachers or social media platforms, all participants were fully informed about the study’s aims, the researchers involved and the intention to publish findings. In an official consent form, all participants were assured that their data would be kept strictly confidential and anonymized. By signing this form, participants gave their consent to use and publish the data for research purposes. To protect the anonymity of the participants and the schools, pseudonyms were developed to refer to the teacher participants following. All data is stored exclusively on a password-protected server space to which only members of the research project have access to. Based on national laws and university statutes and guidelines, it was not necessary to obtain formal ethics approval. The study, however, adhered to the principles of the Declaration of Helsinki.

### Data Preparation and Analysis

In order to maintain quality, the process of analysis was organized following requirements that are considered as ‘standard’ in qualitative research (Golafshani, 2003; Froschauer and Lueger, 2020). Since interviewers often have developed their own view of the course and the ‘correct’ interpretation of the interview, the activities of interviewing and analyzing were separated from each other. However, the interviewing author continued to support this process to check for accuracy and resonance. Following the steps recommended for typological analysis (Hatch, 2002, p. 153), the first and second authors closely read the interview transcripts and agreed on the main typological categories (as well as subcategories) in order to uncover themes and patterns. They developed an initial coding structure that guided the further analysis. The interview material was then divided between them for coding in MAXQDA 2020. Each interview transcript was coded by assigning relevant statements to the typological categories. This allowed for extracting all statements belonging to one or more categories and facilitated analysis. The typological categories aligned to a certain extent with the topics that the interview questions sought to explore. However, due to the open and broad character of the questions, additional themes and categories emerged from the data (e.g., parents’ engagement).

In order to avoid or correct individual perception filters and preconceptions, the code assignment was mutually checked. For this purpose, the two authors met regularly to discuss any divergences regarding code allocation and to reflect on whether the code structure needed to be adapted. Categories and subcategories were systemized, bundled and related to each other in a final step of analysis. Teachers’ narratives were frequently repeated in their responses and mutually corroborated, indicating that data saturation was achieved and that the categories developed were structural features in the teachers’ experiences rather than subjective and biased interpretations by the researchers (cf. Pyett, 2003). As also suggested by Pyett (2003), we presented the results to members of the expert staff of the Board of Education for Vienna, as an additional strategy to assure the study’s validity. These experts had extensive insights into the organization of schooling during school closures and provided additional confirmation that our findings were representative of the situation as they experienced it.

The entire analysis process was accompanied by memo-writing, i.e., writing down the thoughts that occurred during the analysis.

## Results

This section starts with teachers’ descriptions of the organization of learning for GLSC students during the first school closure and the main perceived barriers regarding ensuring these students’ continued learning. The second part focuses on the second school closure and the new barriers and opportunities that emerged in this situation.

### First School Closure

The first school closure started abruptly in March 2020. Teachers and school leaders received the information about the planned school closures along with the rest of the public, during a government press conference on the afternoon of Friday March 13. Although the closure was first announced as starting on Wednesday the 18th, schools were effectively closed from Monday the 16th. Our data collection sought to uncover how home-based learning was organized for students in GLSC during this time.

#### The Organization of Home-Based Learning for Students in German Language Support Classes

All teacher participants reported that schools were caught off guard by the abrupt decision to close schools in March and, unsurprisingly, not at all prepared to support home-based learning for GLSC students. The situation in Austria was much like everywhere else: distance learning provision primarily consisted of ‘learning packages,’ i.e., printed learning material in folders (Helm et al., 2021). All teachers reported that there was very little time to collect worksheets and apart from one teacher, all stated that they had no time to differentiate the learning material according to students’ diverse needs. The folders were sent by mail or picked up by parents. Some teachers reported that they even delivered them to students’ homes.

The teachers revealed that GLSC were more-or-less discontinued during the first school closure and that German language support was not considered as a ‘priority.’ The students’ main classroom teachers were made responsible for providing their learning. This meant that GLSC students were no longer “pulled out” into separate courses but were expected to fully participate in distance learning with the students in their main classroom. It was these main classroom teachers – and not the GLSC teachers – who mainly corresponded with students’ parents and organized the weekly schedules and worksheets for the students. The role of GLSC teachers was mainly limited to supporting the main classroom teacher in three main ways: by providing some extra materials; regularly talking conversationally to GLSC students on the mobile or via video conference; and helping GLSC work on tasks in the learning packages.

Despite not having much direct contact with their students, many GLSC teachers (14) reported that they prepared their own additional language support materials for them. These learning materials, however, were considered as extra-curricular and voluntary, so that students would not be overwhelmed with extra work beyond the main classroom work, or by an extra support person making contact with them. This is why some teachers (3) provided additional material only for students who were already more advanced German language speakers and therefore needed less support from parents or teachers to be able to do the tasks. In addition to print materials, about half of the teachers also provided online materials (e.g., audio files, YouTube videos, online games, and quizzes) with opportunities to experience German in context. The online resources were made accessible via the schools’ homepage, email or a learning application.

While many teachers reported that they were engaged in supporting the main classroom teacher with distance learning to a certain extent, there were a few (5) who reported that they were hardly or not engaged at all in this process. The main explanation provided by these teachers was that they considered distance learning with students with beginner’s skills in German language as not at all feasible. They argued that their students would not understand the instructions on the worksheets and their parents would not be able to help them. Thus, these teachers thought that they would not be of any use to their students, as can be seen in this extract:

… distance learning was absolutely irrelevant for me as a teacher of German language support classes, because I have many, many, many children from the first two basic levels in my class and we agreed in our school location that we would only prepare print material that the children can handle as well as possible on their own. And so it was clear to me that as a teacher of a German language support class I could not provide anything for the children, because I had many children there who could not read. Because I had many children in my groups where I simply knew that the parents could not help either as far as German was concerned. And since we did not do any online teaching or anything like that, I actually took myself out of the planning as far as distance learning was concerned. (Teacher_1).

Other teachers tried to provide additional support for their students by sending them online material. They, however, could not tell whether the children worked with the materials because they had no contact with them:

And all I could really do was stand there and watch and not really provide any support, because the class teachers had the most work to do (…) I then formed a language team with my colleague who also does German language support and then we sent audio recordings by email. (…) [But] the children completely slipped away from me. (Teacher_3).

The feeling of not being able to support the children sufficiently and the worry that the school closure would have a negative impact on their further school career are described by some teachers as emotionally challenging:

… your heart just breaks because they fall behind. (Teacher_9).
So personally, as a teacher, I wasn’t doing well because I knew I couldn’t do what I was supposed to do as a teacher. (Teacher_3).

While the vast majority of teachers interviewed experienced it as very difficult or impossible to organize distance learning for GLSC students, with online teaching not considered an option for most of them, there was one teacher who reported that she had successfully offered online teaching to all 10 of her first grade and two second grade students in GLSC. She reported that in the second week of school closure, she started to provide individual online teaching for each student for 10 min a day, four times a week via WhatsApp or Skype. For two students who were newly arrived in Austria and who had very basic German language skills she extended the online session to a half an hour per day. The session did not only focus on conversation practice, but they also worked together on vocabulary and grammar in order to prepare the students for the MIKA-D test as well as possible.

Then I thought, well, I could actually do something and I thought about a concept and contacted my colleague who was the main classroom teacher (…) and suggested to her that I work with the children who are in the German language support classes and courses daily via WhatsApp video. I then also did that. (…) I had to prepare them for the MIKA-D test and I did grammar and vocabulary with them. I had all the material on my computer (…) so I was able to share the screen with them and ask them what it was. I made sentences with them and exactly those sentences that they needed for the test, because I knew it could come shortly afterward, yes. (Teacher_17).

There did not appear to be anything particular in this teachers’ training or context that made it possible for her to reach GSLC students when the others could not; however, this teachers’ motivation, ingenuity, and perseverance stand out as an exceptional case in the data.

#### Barriers Teachers Perceived

The way distance learning was organized for students in GLSC during the first school closure – as outlined in the previous section – was perceived to present numerous barriers and risks to these students, which are described in the following sections.

##### Language Barriers: Communication Between Teachers and Students

About half of the teachers interviewed reported that they regularly tried to speak briefly with their students on the phone, either by mobile phone or video conference, in order to provide them support with the learning packages, to stay in touch and to encourage the children to keep speaking German. Since most of the students have beginner-level German, communication with them was perceived as even more difficult due to the spatial separation. Communication via these means prevented the use of gestures and facial expressions to support communication (“communicating with hands and feet”), a circumstance that was perceived as a particularly strong barrier in the provision or support. The decision to organize distance learning for GLSC students in the same way as for ‘regular’ students resulted in a further barrier, since often they did not understand task instructions:

In my German language support class, I always explain the assignments to those who do not know German very well, using hands and feet, facial expressions and gestures, and I don’t know what else. And just the worksheets alone, most of the children do not understand the instructions, that is the main problem. They do not know what they have to do. It quickly became clear that they did not understand the information. (Teacher_15).

Some teachers reported that they tried to explain the task instructions by having the students send photos of worksheets they did not understand via text message. They then either gave assistance via the chat function or explained on the phone what the students had to do, which was described as very challenging.

While this was a strategy that many of them used, one teacher reported that she stopped direct communication with students because she experienced it as too exhausting for her and the students:

And then I talked to the students on the phone, but then we quickly let that go, because for everyone, we also tried WhapsApp, but that was so unbelievably exhausting for everyone involved (…) We are at a stage of ‘These are the trousers, these are the green trousers, What color are the trousers? The trousers are green.’ Well, how am I supposed to do that on the phone with the kids … (Teacher_8).

While communication with their students was considered as the main barrier for the provision of distance learning, one of the teachers interviewed reported using professional support from an external language expert who could communicate with the students and their parents in their home languages:

… at that time, I was lucky enough to have this MIKA team at the schools – I do not know if you know it? And there was someone who, thank God, knew at least two languages besides German, and that was a huge help. Without him, I would not have known how to talk to the parents, because I often told him or wrote to him to at least ask how the children were doing, how they were coping, because if I had told the parents, that would not have been possible, at least with those parents who really did not understand anything [in German]. (Teacher_12).

This example of using external language support was the only one found in the dataset.

##### Access to Technology and a Supportive Learning Environment

In addition to language barriers, teachers perceived that many households lacked necessary resources, such as adequate technical equipment with laptops, tablets, mobiles, and (stable) internet. According to them, the restricted provision of technical resources was one of the main barriers for engaging students in online learning:

These children have no laptops, no tablets at home, they have mobile phones, but they do not have their own mobile phones at that age, so they use their dad’s mobile phone. (Teacher_7).

Because it was recognized that some children did not have adequate access to technology at home, the Ministry of Education planned to issue laptops to students in need. However, a further issue that was raised by a few of the participants (4) was that these promised laptops were not distributed to students in GLSC:

We tried to equip the children with equipment. Again and again, we were told that there are laptops, we will get equipment, and we received nothing. We have received nothing until this day. Now in the second lockdown it was asked again and again who needs and who wants, who still needs something. Unfortunately, we have not received anything yet. (Teacher_7).

One teacher even reported that she paid with her own money toward mobile phones for some of her students:

So I paid toward the mobile phones with my personal money and gave [the students] the mobile phones. Used mobile phones, of course. I paid toward mobile phones for three to four children and installed the programs on them. (Taeacher_15).

In addition to access to technology, the students’ living conditions were perceived as another barrier to home-based learning by several teachers. They reported that many of their students do not have well-equipped learning spaces. For example, there were students who did not have their own desks. One teacher reported that some of her students would work on their beds. If technical equipment was available, it often had to be shared with other siblings or parents. Furthermore, teachers reported high noise levels, as there were often many family members in a confined space, which meant that talking on the phone with the teacher was even more difficult as there was no space where the children could talk without being disturbed:

As we know, the living situation is such that the children cannot talk on the phone in peace and quiet, so we let that go. (Teacher_8).

A few teachers who personally brought the ‘learning packages’ to the students’ home or who talked to them via video conference mentioned that they gained insight into the poor living and learning conditions of their students and that this was emotionally stressful for them:

Personally, it was a bit upsetting, because you also get to know the living conditions of the students, in what desolate living conditions they sometimes live. Personally, it was a bit of a challenge to get to know the children from a different perspective than when they only come to school. (Teacher_15).

Some teachers also reported that some students were also very inhibited or embarrassed to talk to their teacher on the phone, especially when their parents were sitting next to them and listening.

##### Parental Support and Engagement

Teachers reported that a further challenge was that many parents were not able to support their children’s learning. The perceived reasons for this were that parents did not understand the task instructions or did not have time to support their children’s school needs due to their working conditions. While support from parents was a well-known challenge for most families during this period, many of the teachers in this study perceived of their students’ parents as being disengaged or not interested in the educational success of their children. This topic took up a lot of space in all the interviews, which is particularly interesting given that there were not any questions that specifically focused on the engagement of parents in the interview schedule.

The teachers’ main criticism refers to the parents’ insufficient knowledge of German or that they did not spend any time practicing German with their children at home.

So the parents are also very important and that the parents accept what the teachers prescribe and what they recommend. That they also have to practise with the child at home. I don’t understand it either, we also have parents who have an older child and who still don’t know any German (…) I don’t understand that at all. (Teacher_2).

While it was clear that many parents did not have the German skills to support their children with their school work, teachers criticized them for a lack of ingenuity and commitment. For example, one teacher pointed out that parents did not use translation applications to understand worksheet instructions:

There is also zero support from the parents. It is not what you would expect. If the parents were extremely engaged, they could also enter the text, the information from the worksheet into Google Translator and translate it themselves into their mother tongue and explain the information to the child. But the parents are not so engaged that they do that and some of them have seven or eight children at home, so they do not do that. (Teacher_15).

Teacher participants also reported that the parents did not create a regular, structured routine for the children. The teachers reported that the children often stayed up late, playing computer games or surfing the internet. They therefore did not get up early in the morning and did not get to their school work until late morning or early afternoon.

It is also difficult because the schedules, the daily rhythms shift. […] if parents do not pay attention, the entire daily routine shifts and I only notice this when the children log on to the chat at eleven. So they no longer have a real daily routine, everything shifts. They no longer do their homework and learning at the time when their attention is best, but at some point when it occurs to them. (Teacher_17).

According to the teachers, this problem continued when the students had to return to school in shifts after the first school closure. There was little understanding expressed by the teachers for the difficulties of managing this situation, particularly for the socio-economically disadvantaged in precarious living situations.

### Second School Closure: Urgent Need to Change Strategy

The overall message coming from teachers was that the first school closure had a detrimental effect on GSLC students’ language development. Many reported that they had the feeling that they had to start from scratch with many students when they returned to school in mid-May 2020.

Not everyone, but many students, were starting from scratch again. So, all the everyday phrases like ‘Can I go to the bathroom?’ were simply no longer there. (Teacher_13).

The negative experiences during this period and its impact on students’ language learning made clear that further periods of home-based learning for GLSC students should be avoided at all costs. This perspective was in line with new regulations from the Ministry of Education to encourage students who attend GLSC or who have special educational needs to stay in school if possible (BMBWF, 2020b):

The conclusion for us at the school was that if there is another lockdown, then we will definitely have to have these students in school. And that was exactly what was planned anyway, what [the Minister of Education] said, that these children must come regardless (Teacher_7).

Thus, during the second school closure in the 2020/2021 school year, teachers reported that they invited the parents to send their children to school – an offer that was accepted by the vast majority.

#### The Organization of Learning for Students in German Language Support Classes

In general, the teachers perceived the second school closure as being better organized and felt they were better prepared to serve their students’ needs. At that time, school was organized so that there was ‘supervision’ offered for 4 h a day for the students who attended school, which included those whose parents could not take care of them at home, those in GLSC and those in special needs classes. School-based time (20 h per week) was labeled ‘supervision’ because, officially, real ‘teaching’ was not supposed to be undertaken in order not to disadvantage students learning at home. Students in school-based supervision were supposed to be supported while working on the learning packages that had been prepared by their main classroom teacher. They were not necessarily grouped with their normal classmates nor allocated to their classroom teacher (who was also in charge of providing online learning sessions for the home-based students). According to the interviews, schools were relatively autonomous with regard to how school-based supervision was organized. This autonomy also applied to how language support for GLSC students was provided. Based on teachers’ accounts, two models emerged that describe the majority of cases:

1. Targeted German language support was offered within GLSC students’ main classroom or in a separate room for up to 6 h per week.
2. German language support classes were created, as in pre-pandemic times, in which teachers offered language support for up to 15 h per week.

A few teachers (2) reported that students received general learning support during ‘supervision time,’ but no targeted German support. One teacher reported that at her school GLSC students were not encouraged to attend school.

Overall, the teachers perceived the second school closure as a positive experience for their students in terms of language learning because they could support them more intensively since there were far fewer students present at school than usual:

For me personally, it was positive with my German language support class, because we had all the AO [extraordinary] children there, that is, all the German language support class and the pull-out course children, who were with me undisturbed for 15 h in that case, had no other work, no sports, no excursions in between. So, it was really my class for 15 h a week. The children were much more used to it, it was less disruptive, you could get through a week’s material properly. So, paradoxically, I thought it was good for my children. (Teacher_6).

#### Barriers Teachers Perceived

Even though the teachers report that they were able to provide high individual support to GLSC students during the second school closure, Corona protection measures such as social distancing and wearing mouth-nose protection made it near-impossible to implement learning methods that the teachers consider central to language learning. For example, singing was not allowed; students could not be involved in games or group work; and the use of materials like balls and building blocks was not possible because students were not allowed to touch learning materials. Moreover, certain activities such as sports or singing not taking place meant that some students no longer had opportunities to demonstrate their talents and gain a sense of achievement:

The fact that sports lessons and music lessons are being dropped, that is, subjects where the children could distinguish themselves, is of course also difficult for their self-confidence. Because they are the same when they play football. And if you have a beautiful voice, if you could sing beautifully, that all falls away, which makes it even worse for the children, in my opinion. (Teacher_8).

Not only had the learning materials and activities become more limited from the teachers’ perspective, external support also fell away. In particular, learning mentors and reading mentors who volunteer to support the schools were no longer allowed in school, meaning that important learning resources for the students were no longer available:

The reading mentors are not allowed to come (…) these are aids that have been very gratefully accepted by children with a non-German mother tongue. Because they can devote themselves much more to the child in individual work than the class teacher can do in the large group. (Teacher_8).

Thus, the decision to allow GLSC students to attend school throughout the school closure offered some possibilities for German language support to be continued. In a few cases, the school set up seemed to allow better individualized support for students to focus on German learning. However, other restrictions which prevented the students from engaging in activities with fluent German speakers meant that these opportunities were still highly constrained by the circumstances.

## Discussion

Based on the findings of this study, the implementation of home-based learning for students in German language support classes (GLSC) during the first school closure can be classified as what Hodges et al. (2020) refer to as “Emergency Remote Teaching.” As indicated by other studies (e.g., Kim and Asbury, 2020; Schleicher, 2020), teachers reported that their schools were unprepared and overwhelmed by the situation. This is unsurprising given the circumstances. What was surprising was that the current study suggests that the provision of language support for GLSC students was discontinued and not perceived as a priority during the first school closure. It is not clear whether this decision was made autonomously by schools or was a top-down decision from the Board of Education or the federal Ministry. Reportedly, this strategy was chosen in order not to overwhelm students by too much work or by conflicting messages from different teachers. Ironically, however, the decision to include GLSC students in the main class – the very classes that they are pulled out of in ‘normal’ circumstances – seemed to contribute to the likelihood that they were under-served or overwhelmed. Because GLSC students were made the full responsibility of their main classroom teacher, they received the same, undifferentiated learning packages as their ‘regular’ peers. They were also put under the main responsibility of their classroom teacher, who was not as aware of their German learning needs as their GLSC teacher and, moreover, could not provide differentiated support. Teachers reported that students often could not understand task instructions, which was found in other newcomer education contexts (cf. O’Connell and Lucić, 2021).

In order to get around the challenge of providing ‘tacked on’ support to the main classroom teachers’ work, many GLSC teachers in this study reported that they provided students with additional print and online material directed at German learning during school closures. These activities, however, were only offered as extra, non-compulsory, support, in addition to students’ ‘regular’ class work. Moreover, these tasks were rather not aligned with mainstream classwork, and thus did not necessarily directly support students’ needs. The extent to which this was the case seemed to depend on how well the GLSC was incorporated into the mainstream school environment and how well the GLSC teacher communicated with the classroom teacher.

While video conferencing was used with many students in mainstream classes during school closures, this was less likely to be used with GLSC students. With one exception, the majority of the teachers considered it as hardly or not at all possible to offer language provision via distance learning, although some teachers did contact students for regular, brief chats. Some attempted this for a while, but gave up due to the feeling of not being able to provide anything useful for their students. They reported feeling completely overwhelmed by the situation and that their efforts were futile and more likely to frustrate or inconvenience their already struggling students. The lack of strategy, support and technology provided them with too many obstacles to persevere with support on their own initiative. The data set, however, also pointed to teachers who went above and beyond the call of their duties by delivering materials to students’ homes, having regular phone and video chats with students, buying students phones with their own money and engaging the support of language experts to communicate with parents. Such practices, however, were only singularly reported in the data. The majority of participants did not experience any central support or sharing of activities or materials and practices for serving these students between schools. There is also a clear need for the development and use of adaptive and contemporary digital tools, materials, videos for German language learning, and further training opportunities for teachers must be driven forward (see Ferlazzo, 2020; Sugarman and Lazarín, 2020). The ineffectiveness of distance learning during the first school closure led to most GLSC students being invited back to school during the second school closures.

This study revealed that teachers’ greatest perceived barriers for providing distance learning for GLSC students during the first school closure were beyond their control and rooted in the sphere of students and their families. These barriers were their students’ low language levels, insufficient access to technical devices (notebooks, tablets, smart phones, and internet connection) and the perceived disengagement of parents or lifestyle practices that are seen as obstructive. Regarding access to technology, at the system level, it is not clear why students were not provided with technical resources, as this would have surely helped them to better cope with the challenges of home-based learning (Huber and Helm, 2020, p. 56). In other studies, it was found that students from socioeconomic disadvantaged background are often viewed as being less able to participate in online learning due to inadequate digital skills (e.g., Bremm and Racherbäumer, 2020; Di Pietro et al., 2020; Uro et al., 2020), so this perception might have contributed to the perceived challenges reported in this context.

In contrast to the perceptions of teachers in this study, findings from the school barometer (Huber and Helm, 2020) provide important insight: additional technological equipment as well as parental support was only found to have a weak effect on students’ home-based learning. What was found to be much more important for students from disadvantaged backgrounds was personalized learning support, since they often show lower metacognitive learning strategies, which are particularly necessary for self-directed learning and the organization of daily routines (see also Bremm and Racherbäumer, 2020; Uro et al., 2020). Teachers in this study rather reveal that GLSC students were not provided with sufficient personalized learning support during periods of home-based learning, due to the way that the courses were organized.

Research undertaken elsewhere found that one of the benefits of home-based learning was that teachers became more interested in the private circumstances of their students, and were better able to consider them for planning their lessons (Schwab et al., 2020). This, however, was not the case for GLSC teachers, who sometimes even reported being distressed when confronted with their students’ home environments. In fact, findings suggest that teachers hold some deficit perspectives toward students and their families. It was reported that parents often could not linguistically grasp the expectations put on their children and how they could support them in fulfilling them. This is in line with other studies that have shown that teachers often perceive the parents of minority-language students as not caring about their children’s education, whereas these parents often have opposing views (see also Di Pietro et al., 2020, p. 27; Gogolin, 2020, p. 180; Yilmaz, 2021). Only a few teachers interviewed seemed to recognize the socio-economically challenges that these families were facing and to express empathy with their difficult situation due to space constraints, etc. Instead of reflecting on the underlying causes of the parents’ behavior, most of the teachers blamed parents and in doing so shifted the “conflict to a moral level” (Nairz-Wirth and Feldmann, 2016, p. 128). Peralta (2019) reports that teachers regularly underestimate the engagement of parents from ethnic minority backgrounds and their contribution to their children’s learning due to a lack of adequate teacher education and training to be enabled to establish relationships between school, family and community.

Based on the teachers’ accounts, students from GLSC were highly affected by distance learning, especially because of the high lack of contact with the German language. This continued to be a challenge during the second school closure, although some schools enabled a situation in which GLSC teachers could work directly with their learners, using much of their time in school for language-supportive teaching. This ‘teaching,’ however, had to be masked as ‘supervision’ and was hampered by Corona measures which did not allow for many active and creative learning methods.

Taken together, the way that GLSC are organized along with commonly held deficit perspectives about students’ and their families might have negatively influenced teachers’ self-efficacy and professional action. It may have prevented teachers and schools from overcoming the perceived ‘lack of fit’ between the schools’ requirements and students’ varied learning conditions influenced by their socioeconomic status (cf. Bremm and Racherbäumer, 2020; Schwab et al., 2020).

Interviews with GLSC teacher raise many questions about why there was not more creative, proactive support for a group of students who were clearly identified as being failed by the system: why was the use of translators only reported in one exceptional situation? Why were mother tongue teachers not drawn upon as resources to communicate with GLSC students and their families during school closures? Other potential support (e.g., tutoring) could have come from pre-service teachers as recommended by Gogolin (2020). Another example of good practice is the project “Homework Mentorships” in which students with beginning skills in the language of instruction received intensive homework support via text and online conferencing from mentors during distance learning (cf. O’Connell and Lucić, 2021).

Finally, and crucially, given the widely accepted perception that GLSC students had been further disadvantaged and excluded by school closures, with teachers in this study nearly unanimously reporting that students’ competences in speaking and writing German deteriorated dramatically, why were there not more accommodations for these students? How can the Ministry’s decision to demand that the MIKA-D test be implemented as usual in June 2020 be justified? This is problematic not only from a humane and pedagogical point of view, it raises also legal questions. As is the case with special education, the labeling-resource dilemma also exists in the realm of German language support: In order to be legally entitled for language support (15 h a week in primary school), students must be negatively diagnosed by MIKA-D test. Due to the first school closure and the way distance learning was organized, many GLSC students did not receive the language support they were legally entitled to. Still, they were subjected to the MIKA-D test with far-reaching consequences for their further educational career and psycho-social health (e.g., grade repetition and stigmatization).

## Conclusion and Limitations

Even though the pandemic affected all stakeholders involved in everyday school life, at-risk students like students with low or emergent skills of the language of instruction have been hit particularly hard (OECD, 2020). The results presented in this article underpin the statement from Schleicher (2020, p. 4) that “those from disadvantaged backgrounds often remain shut out when their schools shut down.” This study shows that the organization of GLSC in Austria contributed to the failure of German support to students during the pandemic and it reveals that thought needs to be given to the development and expansion of a suitable infrastructure to facilitate equitable quality in learning for all children, whether during exceptional or ‘normal’ situations.

This study provides first important insights into the situation of children who attend GLSC in primary schools in Vienna. As with any empirical study, there are limitations. The population of teachers interviewed is relatively small. However, given that participation was during a time where many teachers were experiencing high levels of stress and higher than normal workloads, the insights of 18 teachers working in this system in Vienna is valuable. Moreover, given that even with this relatively small sample, a saturation in findings was reached, it can be concluded that these teachers’ reports give important insight into their experiences of trying to teach students with emergent German during school closures. Although it would have been most illustrative to capture the experiences of GLSC students during this time, restrictions due to COVID-19 made it nearly impossible to conduct interviews with these students and their parents. Moreover, given the separatist nature of the GLSC system, the views of teachers and students in the mainstream classroom might be considered in future explorations of student inclusion and development. Such work could also focus more specially on learners’ language development and/or psychological factors such as wellbeing and inclusion.

## Data Availability Statement

The original contributions presented in the study are included in the article/Supplementary Material, further inquiries can be directed to the corresponding author.

## Ethics Statement

Ethical review and approval was not required for the study on human participants in accordance with the local legislation and institutional requirements. The patients/participants provided their written informed consent to participate in this study.

## Author Contributions

KS organized and supervised the field work (conduction and transcription of interviews). Furthermore, she supported the interpretation of data and was strongly involved in the literature review. MG was responsible for the data analysis and discussion and contributed to the literature review. EE was also responsible for the data analysis and strongly contributed to the discussion and the literature review. SS supported the organization of the field work and interpretation of research findings. Furthermore, she conceptualized the literature review. All authors contributed to the article and approved the submitted version.

## Conflict of Interest

The authors declare that the research was conducted in the absence of any commercial or financial relationships that could be construed as a potential conflict of interest.

## Publisher’s Note

All claims expressed in this article are solely those of the authors and do not necessarily represent those of their affiliated organizations, or those of the publisher, the editors and the reviewers. Any product that may be evaluated in this article, or claim that may be made by its manufacturer, is not guaranteed or endorsed by the publisher.

## Footnotes

1 Differences exist between German Language Support Classes (GLSC) and pull-out courses in terms of structure and length; however, the teachers and the provision in both are often the same. Therefore, for simplicity’s sake, the acronym GLSC is used in the remainder of this article to refer to both.
