# Supplementary material for: Teaching Multilingual Students During the COVID-19 Pandemic in Austria: Teachers’ Perceptions of Barriers to Distance Learning
Source: Front Psychol. 2022 Mar 10;13:805530. doi: 10.3389/fpsyg.2022.805530 (PMC8960454; doi:10.3389/fpsyg.2022.805530)
Supplement: Supplementary file 1 [file Data_Sheet_1.docx]

**Supplement material: Original interview passages from teachers who work in German language support classes in the city of Vienna, Austria**

**First interview passage:**

Für mich war‘s so, dass das Distance Learning für mich als Deutschförderklassenlehrerin absolut irrelevant war, weil ich in meiner DFK viele, viele, viele Kinder aus den ersten zwei Grundstufen hab und wir bei uns in unserem Standort uns darauf geeinigt haben, dass wir nur Print-Material vorbereiten, dass die Kinder so gut wie möglich alleine bewältigen können. Und damit war‘s für mich klar, dass ich als Deutschförderklassenlehrerin den Kindern im Prinzip nichts bereitstellen kann, weil ich viele Kinder da hatte, die nicht lesen können, weil ich viele Kinder in meinen Gruppen hab‘ wo ich einfach weiß, dass auch die Eltern was Deutsch betrifft nicht helfen können und da wir kein Online-Unterricht gemacht haben oder ähnliches ähm hab ich mich dann auch aus der Planung was Distance Learning betrifft eigentlich ganz raus genommen. (Teacher_1)

**Second interview passage:**

Und ich konnte eigentlich nur danebenstehen und kaum unterstützend wirken, weil die Klassenlehrer das meiste zu tun hatten. (…) Wir haben dann ein Sprachteam gebildet, mit meiner Kollegin, die auch die Deutschförderung macht. Und dann haben wir Tonaufnahmen per Mail geschickt. (…) [Aber] die Kinder sind mir komplett entglitten. (Teacher_3)

**Third interview passage:**

Und da bricht es einem das Herz, weil dass, die bleiben zurück (Teacher_9)

**Fourth interview passage:**

Also mir ging es persönlich, als Lehrerin, nicht gut, weil ich wusste, ich kann nicht das leisten, als Lehrerin, was ich eigentlich machen müsste. (Teacher_3)

**Sixth interview passage:**

Dann habe ich mir gedacht, naja, eigentlich könnte ich schon was machen und habe mir ein Konzept überlegt und habe meine Kollegin, die die Klasse geführt hat kontaktiert (…) und habe ihr vorgeschlagen, dass ich mit den Kindern, die in den DFK sind, täglich per WhatsApp Video arbeite. Das habe ich dann auch durchgeführt. (…) Ich habe sie ja vorbereiten müssen auf den MIKA-D Test und ich habe mit ihnen Grammatik und Wortschatz gemacht. Ich hatte das ganze Material auf meinen Computer, das hatte ich eh schon, und dadurch habe ich dann mit ihnen zum Beispiel den Bildschirm teilen können und habe mit ihnen, ahm, habe sie fragen können, was das ist. Ich habe mit ihnen Satzbau gemacht und genau diese Sätze, die sie dann brauchen für den Test, weil der ja dann kurz darauf kam, ja. (Teacher_17)

**Seventh interview passage:**

Bei mir ist es in der Deutschförderklasse so, dass ich ihnen die Arbeitsaufträge natürlich immer, denjenigen, die noch gar nicht gut Deutsch können, mit Händen und Füßen erkläre und Mimik und Gesten was weiß ich alles, und nur die Arbeitsblätter alleine. Die meisten Kinder verstehen die Angaben nicht. Das ist das hauptsächliche Problem. Sie wissen nicht, was sie bei den Arbeitsblättern machen müssen. Das halt sich dann auch schnell heraus gestellt, dass sie die Angaben nicht verstehen. (Teacher_15)

**Eighth interview passage:**

Und dann habe ich mit den Kindern telefoniert, aber das haben wir dann schnell sein lassen, weil das für alle, wir haben auch WhapsApp Telefonie probiert, aber das war so unglaublich anstrengend für alle Beteiligten. Wir sind in einem Stadium von “Das ist die Hose, das ist die grüne Hose, wie ist die Hose? Die Hose ist grün.” Na, wie soll ich das am Telefon mit den Kindern machen und auch auf WhatsApp telefonieren? (Teacher_8)

**Ninth interview passage**

Und ich hatte damals das Glück, es gibt an den Schulen dieses MIKA-Team ich weiß nicht, ob Sie das kennen, und da gab es jemanden, der Gott sei Dank zumindest, ähm, zwei Sprachen neben Deutsch konnte und das war eine Riesenhilfe. Also ohne ihn hätte ich das, weiß ich überhaupt nicht, wie ich mit den Eltern, weil ich habe ihm dann oft gesagt oder geschrieben, dass er zumindest Mal nachfragt, wie es den Kindern geht, wie sie zurechtkommen, weil wenn ich es dann den Eltern, das wäre nicht möglich gewesen, zumindest mit diesen Eltern, die wirklich nichts verstanden haben. (Teacher_12

**Tenth interview passage**

Also diese Kinder haben keine Laptops, keine Tablets zuhause, die haben die Handys, aber sie haben keine eigenen Handys in meiner Klasse also die haben das Handy vom Papa verwendet. (Teacher_7)

**11th interview passage**

Also ich habe teilweise die Handys von meinem Privatgeld gekauft und habe ihnen die Handys gegeben, also gebrauchte Handys natürlich. Ich habe teilweise für drei bis vier Kinder diese Handys gekauft und habe ihnen die Programme darauf installiert. (Teacher_15)

**12th interview passage**

Und wir haben schon versucht die Kinder auch auszustatten mit Equipment also immer wieder hat es geheißen, es gibt Laptops, wir bekommen Ausrüstung, und wir haben nichts bekommen, wir haben bis jetzt nichts bekommen. Jetzt im zweiten Lockdown wurde immer wieder erhoben, wer braucht und wer möchte, wer braucht noch etwas. Wir haben leider noch nichts bekommen bis jetzt. (Teacher_7)

**13th interview passage:**

Bekannt ist die Wohnsituation so, dass die Kinder in der Regel nicht in Ruhe telefonieren können und das haben wir dann sein lassen. (Teacher_8)

**14th interview passage:**

Persönlich war es ein bisschen aufwühlend, weil man halt auch die Lebensverhältnisse der Schüler kennen lernt, in welchen desolaten Lebensverhältnisse sie teilweise leben. Es war halt persönlich ein bisschen eine Herausforderung sich darauf einzulassen, die Kinder aus einer anderen Perspektive kennen zu lernen, als wenn sie nur in die Schule kommen. (Teacher_15)

**15th interview passage:**

Also ganz wichtig sind eben die Eltern auch und, dass die Eltern akzeptieren, was der Lehrer vorgibt und was er empfiehlt, dass sie eben Zuhause auch mit dem Kind üben müssen. Also ich versteh’ es auch nicht, wir haben auch Eltern, die noch ein älteres Kind haben und die noch immer gar kein Deutsch können (…) Das verstehe ich überhaupt nicht. (Teacher_2)

**16th interview passage:**

Von den Eltern kommt halt auch Null Unterstützung. Es ist nicht so, wie man sich das halt erwarten würde. Wenn die Eltern extrem engagiert wären, könnten Sie auch den Text, die Angaben vom Arbeitsblatt in Google Übersetzer eingeben und selber übersetzen in ihre Muttersprache und dem Kind die Angabe erklären. Aber die Eltern sind jetzt nicht so engagiert, dass sie das machen und haben teilweise sieben, acht Kinder zu Hause. Die machen das halt nicht. (Teacher_15)

**17th interview passage:**

Es ist halt auch schwierig, weil die Zeitabläufe, die Tagesrhythmen sich verschieben. Das gilt bei Regelklassenkindern auch, dass wenn Eltern nicht darauf achten, sich der gesamte Tagesablauf sich verschiebt und ich merke das erst, wenn die Kinder sich erst um elf in den Chat einloggen. Sie haben also keinen richtigen Tagesrhythmus mehr, es verschiebt alles. Sie machen die Hausübung und das Lernen nicht mehr zu der Zeit, wo die Aufmerksamkeit noch am besten ist, sondern irgendwann, wenn es ihnen einfällt. (Teacher_17)

**18th interview passage:**

Man hat schon, nicht bei allen, aber doch bei vielen, wieder eher bei Null angefangen. Also die ganzen Alltagsphrasen wie „Darf ich auf’s Klo gehen?“, die waren einfach nicht mehr da (Teacher_13)

**19th interview passage:**

Und ja, Resümee für uns dann and der Schule war, wenn es wieder zu einem Lockdown kommt, dann werden wir auf jeden Fall die Kinder in die Schule holen müssen. Und das war dann genau das, was eh vorgesehen war, was dann der Faßmann noch gesagt hat, dass diese Kinder auf jeden Fall kommen müssen. (Teacher_7)

**20th interview passage:**

Für mich persönlich, mit meiner Deutschförderklasse war es positiv, weil wir hatten alle AO Kinder da, das heißt alle Deutschförderklassen und Deutschförderkurskinder, die waren ungestört fünfzehn Stunden bei dem Fall bei mir, hatten kein Werken, kein Turnen, kein Ausflug dazwischen. Also es war wirklich fünfzehn Stunden in der Woche meine Klasse. Die Kinder waren das viel gewohnter, es war weniger zerrissen. Man konnte ordentlich einen Wochenstoff durchbringen. Von daher fand ich paradoxer Weise, war das gut für meine Kinder. (Teacher_6)

**21st interview passage:**

Dass Turnunterricht wegfällt, Musikunterricht, also Fächer, wo sich die Kinder profilieren könnten, das ist für das Selbstbewusstsein natürlich auch schwierig, weil beim Fußball spielen sind sie gleich. Und wenn man eine schöne Stimme hat, wenn man schön singen könnte, das fällt alles weg, das ist für die Kinder doppelt schlimm, meiner Meinung nach. (Teacher_8)

**22nd interview passage:**

Die Lesepaten dürfen nicht kommen (…) das sind Hilfen, die bei Kindern mit nichtdeutscher Muttersprache sehr dankbar angenommen worden sind. Weil die sich viel mehr dem Kind zuwenden können in der Einzelarbeit, als dass der Klassenlehrer machen kann in der großen Gruppe (Teacher_8)
